# Epidemic Typhoid Fever*Read before Richmond Academy of Medicine and Surgery, July 14th, 1896.

**Published:** 1896-08

**Authors:** William S. Gordon

**Affiliations:** Richmond, Va.; Professor of Physiology, University College of Medicine, Richmond, Va.; 410 East Grace Street


### EPIDEMIC TYPHOID FEVER.*

BY WILLIAM S. GORDON, M. D., RICHMOND, VA.

Professor of Physiology, University College of Medicine, Richmond, Va.

My design in this paper is not to treat the subject exhaustively, but to present a running commentary on about twenty-five cases of typhoid fever occurring in an institution for boys of which I was at one time the attending physician.

The disease made its appearance early in September, 1895, and prevailed until the close of the year. If dysentery and gastro-intestinal disturbances be included, the whole number of cases would amount to thirty-five or forty. Under the measures adopted, with the intelligent and unremitting attention of a trained nurse, we were fortunate in having no deaths.

*Read before Richmond Academy of Medicine and Surgery, July 14th, 1896.

The first cases owed their origin to the use of polluted well water; while others, occurring later in the epidemic, were due, in my opinion, to the use of water from a polluted stream. The cases developed after the usual incubating period—two or three weeks from the entrance of the poison into the system; and the arguments for the correctness of my views as to the cause of the outbreak are irrefutable. It is needless to discuss this portion of the subject.

I had a hospital erected for the patients, and the directions regarding disinfection and other hygienic measures were faithfully followed by the head nurse.

The grouping of the cases enabled me to observe, to the best advantage, the difference in the course of the disease, the symptoms, and the response to treatment in individual cases; and the demonstration that a common infection may produce many exceptions to the ordinary and classic symptoms of the typical disease was conclusive. I am also further established in my belief that the cases of so-called typho-malarial fever are, in the large majority of instances, cases of atypical typhoid fever, the difference in symptoms being partly accounted for by the concentration of the poison and the susceptibility to it of the individual constitutions.

In most of the cases the invasion was abrupt, resembling that of malarial fever or the grip. Epistaxis occurred in a few cases. Constipation was oftener present than diarrhœa. The highest temperature was $105\frac{3}{5}°$; the most rapid pulse about 130. Headache was common, and either a decided chill, or chilliness, was the rule at the onset. The cutaneous eruption occurred in several cases. Right iliac or abdominal tenderness, with tympanites, was almost uniformly present. The brown, dry tongue was noticed here and there, while marked nervous symptoms—muttering delirium and semi-coma—occurred in a few instances, and active delirium in two. Sweating was observed occasionally, and bronchitis was rarely absent. The fever was, at times, irregular; but the morning remission and afternoon rise were quite constant. In one or two cases the pulse rate was slow and the beat sluggish, and, I might say, distrustful. Serious hemorrhage from the intestines occurred in only one case.

The shortest duration was about ten days—the longest nearly two months. Post-typhoid insanity of a mild type occurred in one instance. Sore feet was a prominent symptom in one case, and was much complained of by the patient.

Several patients had pain in the joints. Obstinate constipation developed in three or four cases, requiring mechanical means for its relief.

Such was the general course of the epidemic.

My routine plan of treatment was to put the patient to bed immediately, reassure and quiet his mind, have his clothing changed and his skin cleansed, order a liquid diet—milk or milk and broth alternately—and quinine to gentle cinchonism if the symptoms of typhoid fever were not sufficiently marked to justify a positive diagnosis.

My experience with quinine leads me to believe that it will almost infallibly cut short a malarial fever, but never typhoid. In the latter disease, I have seen the drug reduce the temperature, as it often does in other fevers; but, on the other hand, I have also seen the temperature rise after its administration. This result may be due at times to the natural course of the disease, but at others it is doubtless owing to the irritating effect of the agent upon some constitutions. My rule, therefore, is to discontinue it if the fever does not yield in several days, and not to resume it until convalescence, when, with some preparation of nux vomica or other tonic it is of great service.

Sweet and buttermilk were the basis of dietetic treatment, especially the former. Buttermilk, or sweet milk, with strained oatmeal gruel, served a good purpose in the cases characterized by constipation and mild symptoms; and in these I also used occasionally steamed eggs, beef extract, and fresh crackers soaked in milk. If the fever rose, I returned to milk alone or milk and broth. When diarrhœa was present, milk and barley gruel, or milk with arrowroot, proved very serviceable; or milk and lime-water alone. In other cases, a pinch of salt in the milk rendered it agreeable, and evidently more digestible. The free use of pure water was ordered. The adaptation of the diet to the requirements of individual cases is, in my opinion, one of the most important measures in the successful management of typhoid fever.

Chloral-thymol was used freely with good effect.

For the fever, sweet spirit of nitre, spirit of mindererus, and tepid baths, were used. The patients were bathed by running oil cloths under them and sponging freely when necessary. These measures sufficed to keep the skin and kidneys active. Constipation was relieved by enemata every other day, or every day, according to indications, with an occasional dose of oil if required. Diarrhœa was untreated except when the passages were more than three or four in the twenty-four hours. Bismuth, or bismuth and paregoric, answered every purpose. The brown, dry tongue and meteorism were in nearly every instance relieved by an emulsion of turpentine, to which the bismuth, or bismuth and paregoric, was added, when required to keep the diarrhœa in check. Salol was employed in several cases, and appeared to act well.

Epistaxis had to be controlled in one case by the injection of a strong alum solution into the nares. Cold cloths to the head mitigated the cephalalgia.

Turpentine stupes were used for abdominal pain. I wish to say a few words with regard to the use of turpentine. There is no one drug which, it seems to me, meets so many indications, and my increasing experience with it leads me to have an increasing respect for its power. I do not claim infallibility for it, but it is a stimulant, a diuretic, an antiseptic, a healer of intestinal ulcers, and of inflamed mucous membranes in general, and a styptic. In my hands, it has rarely failed to accomplish some good—notably in lessening the meteorism and restoring the dry brown tongue to its moist condition.

In many of the cases, dilute phosphoric acid appeared to render the patient more comfortable. It is a mild feeder of the nervous system and a refrigerant. Alarming hemorrhage occurred in one case. The nurse had been prepared for this, and narcotized the patient promptly with paregoric, administering turpentine, withholding food for twelve hours, applying an ice-bag to the abdomen, and enforcing absolute repose of body and mind. Insomnia was met with cold sponging accompanied with stimulation in cases of exhaustion. The bromides acted well on several occasions. I did not have to use opiates, but have used them with beneficial effect in nerve exhaustion and active delirium.

Bronchial symptoms were treated with turpentine plasters. One of the youngest cases had to be fed by enemata for a short while. In this case, and one or two others, the heart was so weak at times that I almost despaired of being able to strengthen it, but the free use of whiskey and strychnine accomplished all that could be desired.

One of the worst cases was that of the young man who assisted the head nurse in her arduous work. He walked into my office with the disease well developed, and was sent to the Virginia Hospital, where, under the skillful attention of Dr. Vaden, the house physician, and the nurses, he had a long but successfully-fought attack of the disease. I do not know whether he disregarded my orders as to the polluted water, or whether he was negligent in handling the patients and received the poison at close range, so to speak. In this case, Cheyne-Stokes respiration was marked for several days, while as much as one-fifth of a grain of strychnine within two or three hours was, on one occasion, with other measures, required to sustain his flagging heart. In this case, also, the meteorism yielded promptly to salol and gave no further trouble. The slowing and strengthening effects of digitalis on his heart were noticeable.

I do not believe that we have any specific treatment for typhoid fever. Hundreds of physicians have hundreds of remedies, but the disease is apt to run its course. The very fact that excellent results are reported from so many different kinds of treatment is sufficient proof that nothing has the same influence for good in typhoid infection that quinine has in malarial diseases. I do not believe, therefore, that typhoid fever is aborted in the sense that the germ is destroyed; but I do believe that its course may be abridged or modified by appropriate treatment—adopted in the early stages, especially—of the disease. Furthermore, there can be little doubt in the mind of the close observer that intestinal antisepsis and elimination are very important elements in correct treatment. This does not imply such a purgation as would tend to irritate an inflamed or ulcerated bowel, nor such an attempt at antisepsis as would be likely to kill the patient and not injure the germ, which is also engaged in the effort to kill.

This epidemic convinces me additionally of the facility which water offers for the conveyance of the typhoid infectious material. It can not be held that the germs may not be borne at short distances in the atmosphere, breathed in and lodged in the alimentary canal. Air can float germs as well as water, but from the very nature of things it can be easily seen how much more liable the system is to be infected by the germs of typhoid fever in water than in air. The rapid multiplication of the germs in water and milk must be borne in mind.

As already previously intimated, the study of this, as well as other epidemics, proves conclusively that one poison does not, in each case, produce necessarily the same set of symptoms. The very opposite symptoms, in typhoid fever, of diarrhœa or constipation, may be noted. In one case, the fever may run a classical course; in another, it may hardly rise above the normal, or else be sub-normal. And the remark applies to other symptoms. Before we finally sanction the name of a new disease—typho-malarial—it must be clearly defined to what extent the symptoms of a typical typhoid fever may be observed.

I do not claim that my methods in the treatment of the present epidemic are superior to those adopted by clinicians of wider experience. In the treatment of any one symptom, various remedies, acting practically in the same way, may be employed. I have laid down only the general lines of therapeusis, as I conceive them, while I reiterate my fixed belief that until a true typhoid germ-destroyer is discovered, we must depend upon careful hygienic measures, intelligent nursing, and judicious intestinal elimination and antisepsis.

*410 East Grace Street.*

---

THE meeting of the American Dermatological Association will be held at the Hot Springs of Virginia, September 8th, 9th and 10th, 1896. Everything will be done to make the meeting a success, and several papers on interesting subjects have already been promised. Dr. White wi l open a general discussion on the subject, "What Effect do Diet and Alcohol have upon the Causation And Course of the Eczematous Affections and Psoriasis."

